# NAALADL1 modulates cellular resistance to Tumor Treating Fields in colorectal cancer

**DOI:** 10.1038/s41698-026-01492-0

**Published:** 2026-05-26

**Authors:** Zhaoran Su, Menglan Liu, Mathias Krohn, Jasmin Ostermann, Sandra Schwarz, Olga Hahn, Marcus Frank, Timo Gemoll, Clemens Schafmayer, Michael Linnebacher

**Affiliations:** 1 Department of Gastrointestinal Surgery, People’s Hospital of Tongling City, Tongling, 244000 China; 2 Molecular Oncology and Immunotherapy, Clinic of General Surgery, Rostock University Medical Center, Rostock, 18057 Germany; 3 Section for Translational Surgical Oncology and Biobanking, Department of Surgery, University of Lübeck and University Hospital Schleswig-Holstein, Campus Lübeck, 23538 Lübeck Germany; 4 Institute of Cell Biology, Rostock University Medical Center Rostock, 18057 Rostock, Germany; 5 Department Life, Light and Matter, Rostock University, 18051 Rostock, Germany; 6 Electron Microscopy Centre, Rostock University Medical Centre, 18057 Rostock, Germany

**Keywords:** Cancer, Cell biology, Computational biology and bioinformatics, Oncology

## Abstract

Colorectal cancer (CRC), the third most common malignancy worldwide, remains difficult to treat in advanced stages. Tumor Treating Fields (TTFields) are a non-invasive therapy that disrupts mitosis via intermediate frequency alternating electric fields, already clinically approved and widely implemented in the treatment of glioblastoma. This study is the first to systematically evaluate the therapeutic efficacy of TTFields in a large panel of low-passage CRC cell lines, representing diverse biological backgrounds. Our results demonstrate that TTFields exert antitumor effects through multiple mechanisms, including microtubule disruption, metabolic stress induction, mitotic aberrations, DNA damage, and activation of apoptotic pathways. Transcriptomic profiling, targeted sequencing, and weighted gene co-expression network analysis identified N-acetylated alpha-linked acidic dipeptidase-like 1 (*NAALADL1*) as a resistance-associated gene. NAALADL1 proved significantly upregulated in resistant lines, and its knockdown via shRNA sensitized cells to TTFields. Mechanistically, NAALADL1 depletion destabilized microtubules and induced G2/M arrest, enhancing TTFields efficacy. Virtual screening further identified Lumacaftor and Bestatin as candidate NAALADL1 inhibitors, which synergized with TTFields to suppress CRC cell growth. These findings highlight significant heterogeneity in CRC cell line responses to TTFields and identify NAALADL1 as a key modulator of resistance, suggesting its potential as a target for improving TTFields-based therapies in CRC.

## Introduction

Colorectal cancer (CRC) ranks as the third most commonly diagnosed malignancy worldwide and remains a leading cause of cancer-related mortality, with a growing incidence among younger populations^1^. Despite notable advances in treatments including surgical resection, chemotherapy, targeted agents, and immunotherapy, the prognosis for patients with advanced or metastatic CRC remains poor, due to therapeutic resistance and distant metastasis^2–5^.

Tumor Treating Fields (TTFields) therapy is an emerging, non-invasive anti-cancer modality that utilizes low-intensity, intermediate-frequency alternating electric fields to disrupt mitotic processes in proliferating tumor cells^6,7^. By interfering with spindle formation and chromosome segregation, TTFields induces mitotic arrest and apoptosis, resulting in selective cytotoxicity toward dividing cancer cells while sparing normal tissues^8^. It has demonstrated significant clinical benefit in glioblastoma multiforme (GBM) and has received FDA approval for use in both newly diagnosed and recurrent GBM^9,10^. Despite increasing application in other solid tumors including mesothelioma^11^, non-small cell lung cancer^12^, breast^13^, hepatic^14^, and pancreatic cancers^15^, the role of TTFields in CRC remains insufficiently characterized. This knowledge gap is further complicated by the pronounced molecular heterogeneity of CRC, which encompasses various consensus molecular subtypes, common oncogenic mutations, and highly variable tumor microenvironment compositions^16–18^.

Low-passage CRC cell lines derived from primary tumors or patient-derived xenografts (PDX) are clinically relevant preclinical models that largely preserve the genetic, transcriptomic, and phenotypic features of the original tumors^19,20^. They retain subtype-specific characteristics and are frequently accompanied by detailed clinical annotation, supporting mechanistic studies, biomarker identification, and drug response profiling^21^.

In this study, we systematically evaluate the therapeutic potential of TTFields in CRC using a panel of low-passage, molecularly characterized CRC cell lines. By integrating phenotypic, transcriptomic, proteomic, and functional analyses, we aimed to define mechanisms of response and resistance and to identify candidate predictive biomarkers. These findings establish a preclinical framework for precision application of TTFields in colorectal cancer.

## Results

### Ultrastructural alterations highlight response heterogeneity to TTFields in CRC cell lines

**Fig. 1:**
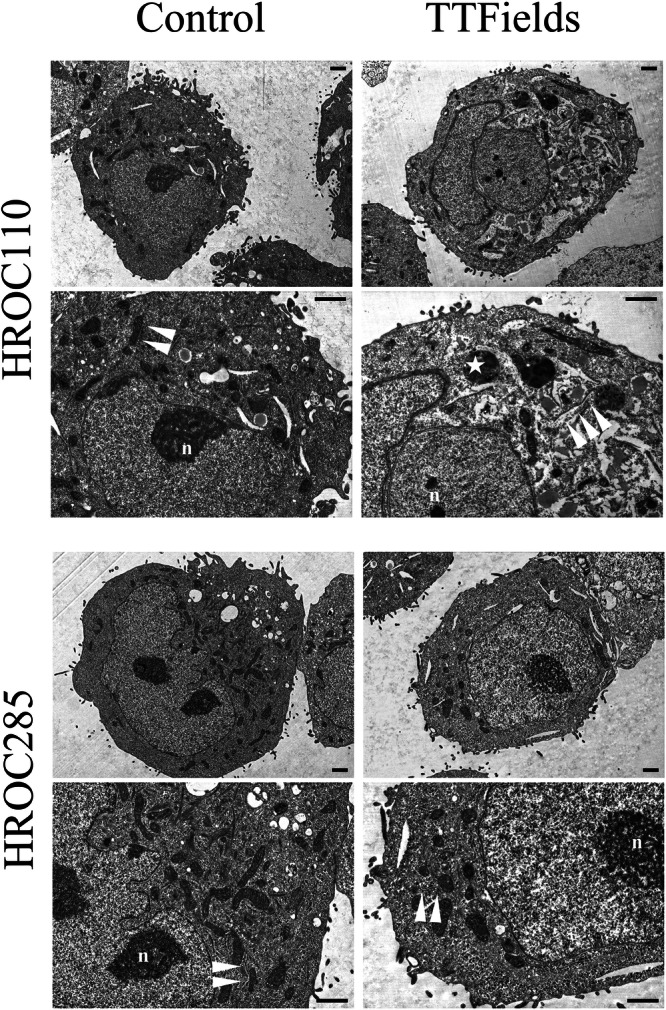
Comparison of ultrastructural changes in CRC cell lines after 48 hours of TTFields exposure at 100 kHz. STEM images of CRC cells, including the control group and cells treated with TTF for 48 hours, are shown. HROC110 cells frequently exhibited significant changes in nuclear morphology after treatment, including granular alterations in the nucleoplasm and nucleolus (n). Notable mitochondrial damage occurred, along with their displacement from the endoplasmic reticulum, accompanied by slit-like vacuolization (arrowheads). These changes may lead to mitochondrial loss and the formation of (auto)phagosomes (asterisks). In contrast, no significant changes were observed in HROC285 cells under the same treatment conditions. Scale bar = 1 µm.

Building on our previous study^22^, which demonstrated that TTFields reduce CRC cell viability in a frequency- and duration-dependent manner, we further investigated early morphological changes to explore potential ultrastructural correlates of TTFields sensitivity. Two CRC cell lines with previously characterized response profiles—HROC110 (relatively sensitive) and HROC285 (relatively resistant)—were selected for scanning transmission electron microscopy (STEM) following 48 hours of continuous TTFields exposure at 100 kHz, allowing assessment of early ultrastructural alterations. STEM analysis revealed striking ultrastructural differences between the two cell lines. Negative control cells of both HROC cell lines showed intact cell and nuclear morphology with well-preserved organelles, and numerous cells exhibited clearly visible pseudopodia, indicating viable cells after detachment. In HROC110, TTFields treatment reduced pseudopodia prominence and induced chromatin condensation, along with irregular nuclear morphology and granular changes in the nucleoplasm and nucleolus. Mitochondrial swelling and damage, along with disruption of the adjacent endoplasmic reticulum and slit-like vacuolization, indicated pronounced cellular stress and organelle dysfunction. In contrast, the HROC285 treatment group exhibited no notable ultrastructural alterations relative to the control group. These findings illustrate the heterogeneity in TTFields responsiveness among CRC models and suggest that early ultrastructural changes may serve as morphological indicators of intrinsic sensitivity (Fig. 1).

### Antitumor effect of TTFields on low-passage CRC cell lines

**Fig. 2:**
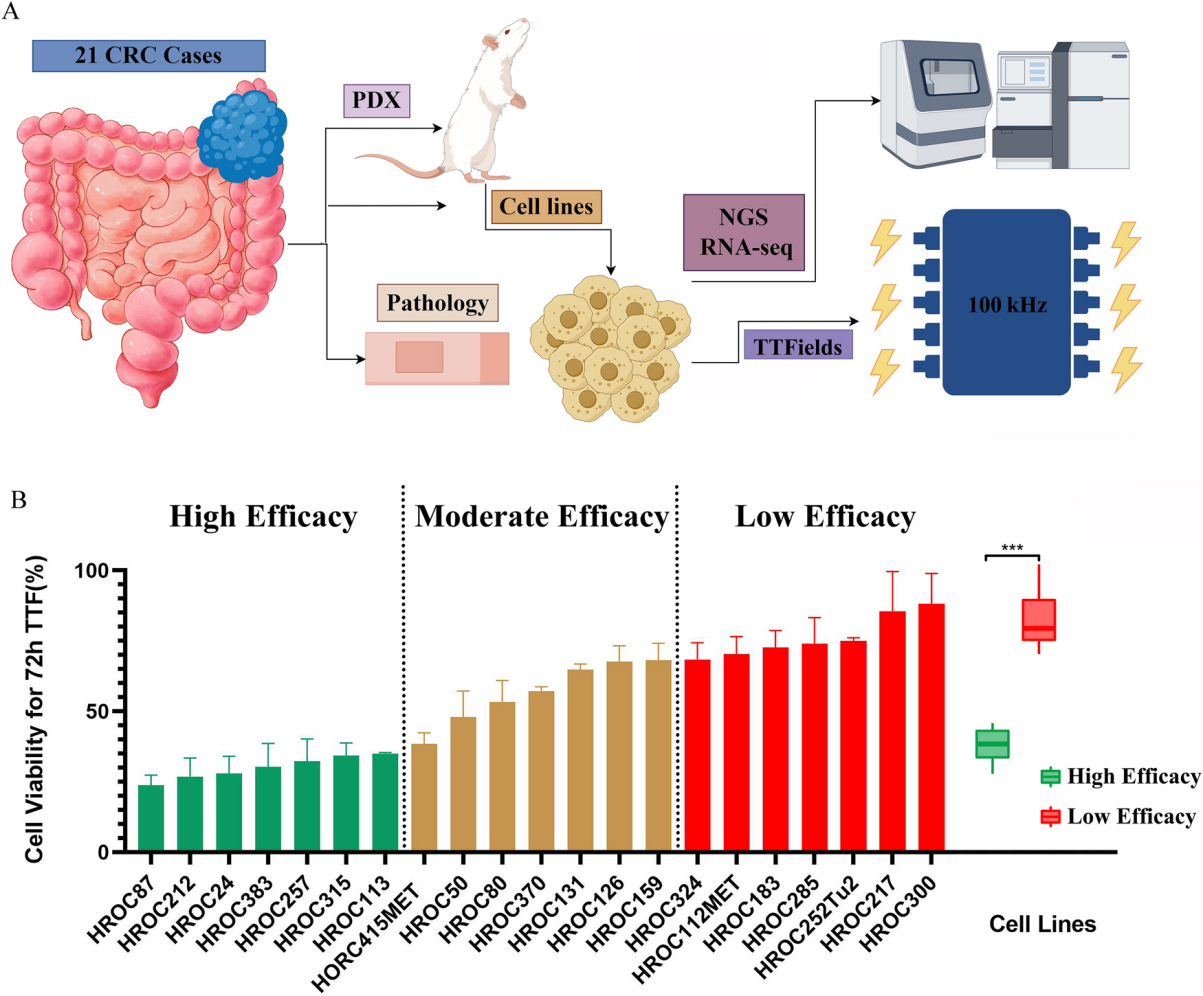
Antitumor effect of TTF on 21 low-passage CRC cell lines. **A** Schematic workflow of the experimental design for assessing the antitumor effect of TTFields on 21 low-passage CRC cell lines. **B** Crystal violet assay results demonstrated the viability of 21 low-passage CRC cell lines after 72 h of TTFields treatment. Cell viability ranged from 23.8% to 88.2%, allowing classification into three groups: high efficacy, moderate efficacy, and low efficacy. Statistical analysis revealed that the low efficacy group exhibited significantly higher cell viability compared to the high efficacy group (*P* < 0.001).

To better characterize the heterogeneity of TTFields response in CRC, we expanded our investigation to a larger and more diverse panel of low-passage CRC cell lines. These 21 lines, established either directly from patient tumors or derived from PDX models, preserve key molecular and pathological features of the original tumors, providing a clinically relevant platform for in vitro testing (Table 1)^20^. All 21 cell lines were exposed to TTFields at 100 kHz for 72 hours—a condition previously identified in vitro (Fig. 2A and Supplementary Fig. 1). Cell viability varied widely after treatment, ranging from 23.8% to 88.2%, reflecting substantial intertumoral variability. Based on post-treatment cell viability, cell lines were ranked and divided into three equal-sized groups representing high-, moderate-, and low-efficacy responses. As shown in Fig. 2B, the low-efficacy group exhibited significantly higher residual viability than the high-efficacy group (*P* < 0.001), underscoring differential sensitivity to TTFields across CRC models.

**Table 1:** The clinical and pathological characteristics of 21 CRC patients

Characteristic	*n* = 21
Age (mean, SD)	63.7 (19.1)
Gender	
Male	5
Female	16
Tumor tissue site of origin	
Right colon	12
Transverse	1
Left colon	3
Rectum	2
Sigmoid	1
Liver	1
Abdominal wall	1
Differentiation	
G2	10
G3	11
UICC stage	
I	2
II	5
III	7
IV	7
Pathological molecular subtype	
spSTD	3
spMSI	9
LS	6
CIMP-H	3
MSI status	
MSI	16
MSS	5

### TTFields exposure is associated with mitotic disruption and apoptosis in CRC cells

**Fig. 3:**
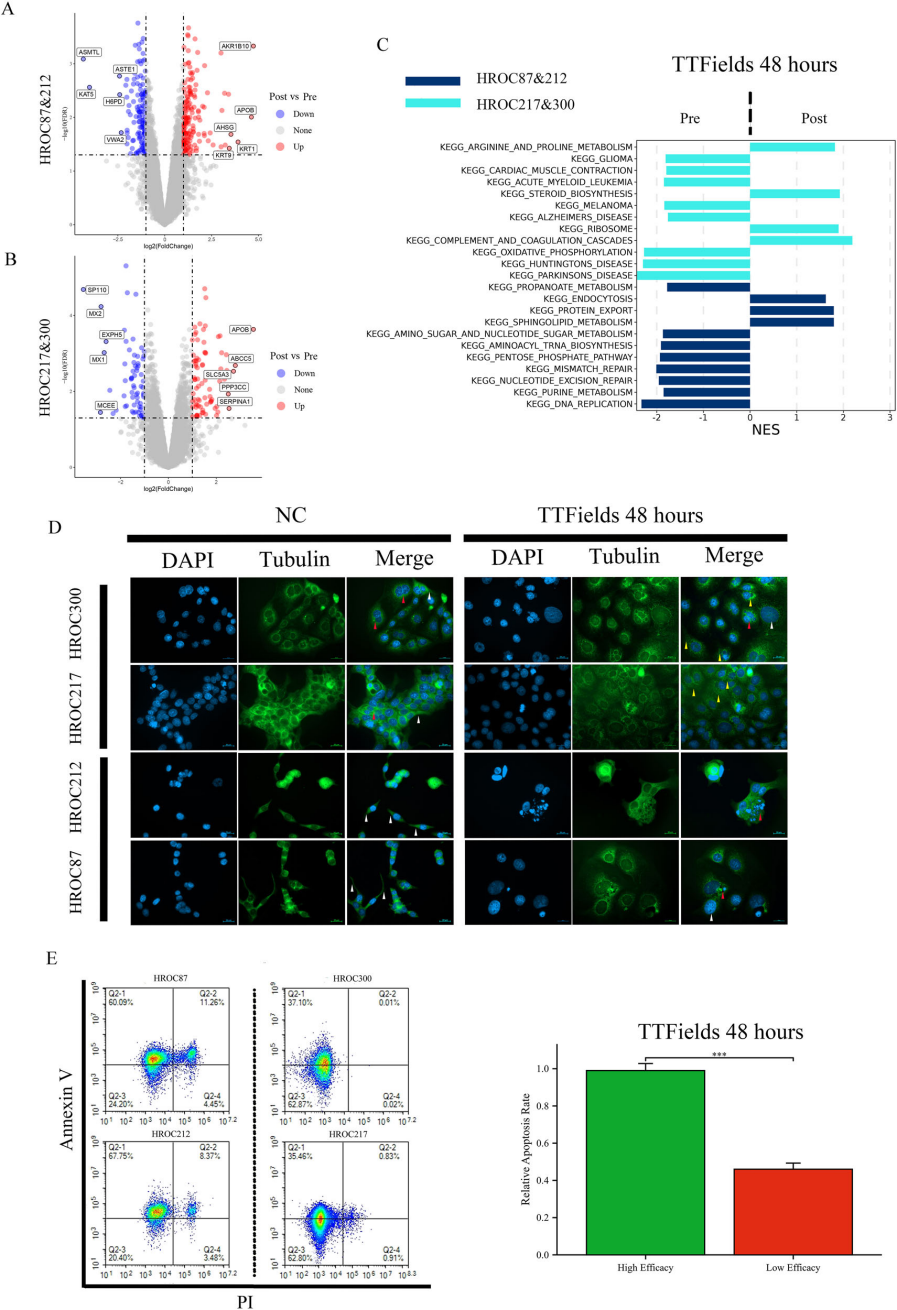
TTFields triggers apoptosis and alters ultrastructure, protein expression, cellular pathways, and microtubule structure in CRC cells. **A** Volcano plot depicting differentially expressed proteins in the most TTFields-sensitive CRC cell lines (HROC87 and HROC212) after 48 h of treatment. Compared to untreated controls, 383 proteins were significantly upregulated and 353 significantly downregulated in TTFields-treated cell lines. **B** Volcano plot depicting differentially expressed proteins in the two most resistant (HROC217 and HROC300) after 48 h of treatment. Compared to untreated controls, 79 proteins were significantly upregulated and 68 significantly downregulated in TTFields-treated cell lines. **C** Functional enrichment analysis of differentially expressed proteins between un- and TTFields-treated cell lines. **D** Immunofluorescence analysis showed that untreated cells had well-organized microtubules with clear polarity (white arrow) and normal mitosis (red arrow), while TTFields-treated cells displayed fragmented microtubules (yellow arrow), abnormal nuclei, and mitotic arrest or apoptosis (red arrow). Scale bar = 20 µm. **E** FC analysis showed significantly higher apoptosis rates in the most TTFields-sensitive cell lines (HROC87 and HROC212) compared to resistant ones (HROC217 and HROC300).

To further explore the mechanisms underlying TTFields efficacy in CRC, we selected the two most sensitive (HROC87 and HROC212) and the two most resistant (HROC217 and HROC300) CRC cell lines based on the screening results. Mass spectrometry (MS)-based proteomic analysis was conducted on these four cell lines following 48-hour TTFields exposure (100 kHz, 24 h/day), quantifying dynamic changes in expression levels of 7479 proteins. Compared to untreated controls, 383 proteins were significantly upregulated and 353 were significantly downregulated in TTF-treated cell lines (Fig. 3A). Functional enrichment analysis revealed that these differentially expressed proteins were enriched in 11 distinct pathways, including propanoate metabolism, endocytosis, protein export, sphingolipid metabolism, amino sugar and nucleotide sugar metabolism, aminoacyl-tRNA biosynthesis, pentose phosphate pathway, mismatch repair, nucleotide excision repair, purine metabolism, and DNA replication (Fig. 3C and Supplementary Data 1). Fewer changes in protein expression were observed in the two most resistant cell lines following TTFields treatment, with only 79 proteins significantly upregulated and 68 significantly downregulated (Fig. 3B). Functional enrichment analysis showed that these differentially expressed proteins were enriched in 12 distinct pathways, including arginine and proline metabolism, glioma, cardiac muscle contraction, acute myeloid leukemia, steroid biosynthesis, melanoma, Alzheimer’s disease, ribosome, complement and coagulation cascades, oxidative phosphorylation, Huntington’s disease, and Parkinson’s disease pathways (Fig. 3C and Supplementary Data 1). Immunofluorescence staining further revealed marked structural abnormalities in CRC cells following TTFields treatment. In the control group, microtubule networks were well-organized with clear polarity, nuclei maintained normal morphology, and mitotic figures were readily observed. In contrast, cells exposed to TTFields exhibited varying degrees of spindle apparatus disruption and nuclear deformation across all four tested cell lines. These features are consistent with mitotic arrest and early apoptotic events. Notably, the TTFields-sensitive cell lines also displayed more pronounced morphological abnormalities compared to the resistant lines, further supporting their differential responsiveness (Fig. 3D). Flow cytometry (FC) confirmed significantly higher apoptotic rates in the two most sensitive cell lines compared to the two most resistant lines following TTFields exposure (*P* < 0.001, Fig. 3E). In sum, these data indicate that TTFields exposure is associated with apoptosis in responsive CRC cells, accompanied by mitotic disruption, metabolic alterations, and DNA damage–related changes.

### Comparison of cell division rate, mutations, and clinicopathological features between high- and low-efficacy TTFields treatment groups

**Fig. 4:**
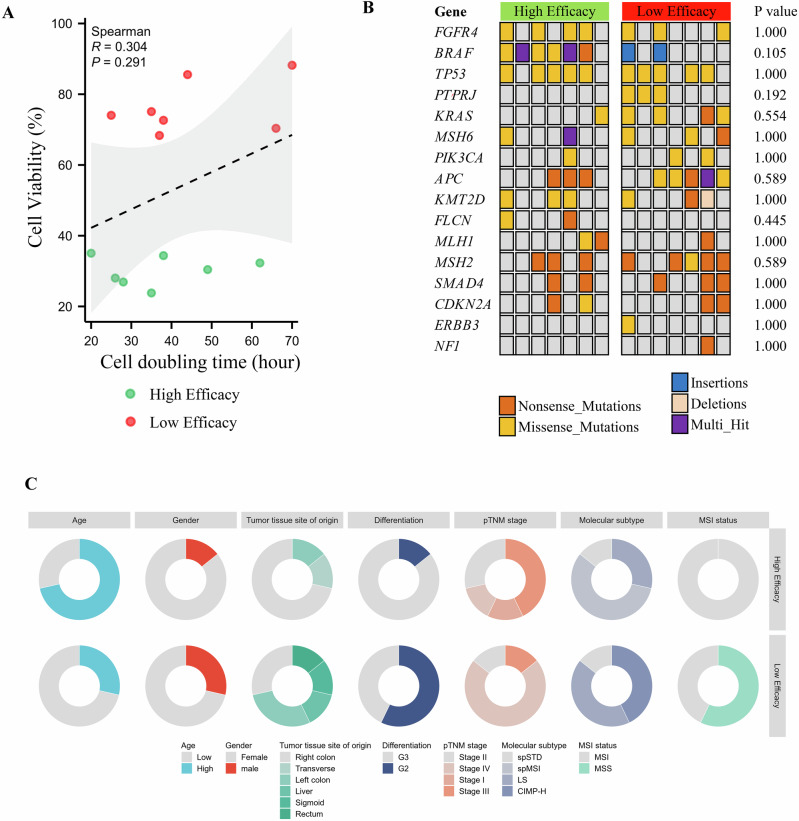
Cell division rate, mutations, and clinicopathological features between high and low efficacy TTFields treatment groups. **A** Correlation analysis between cell viability after TTFields treatment and cell doubling time showed no significant correlation (R = 0.304, *P* = 0.291). **B** No significant differences in the frequency or distribution of specific mutations were observed between the high- and low-efficacy responder groups. **C** Clinicopathological characteristics—including patient age, gender, tumor site of origin, differentiation grade, pathological TNM stage, molecular subtypes, and MSI status—showed no significant differences between the two groups.

To explore factors influencing TTFields treatment efficacy in CRC cells, cell division rates, genetic mutations, and clinicopathological features were compared between the high-efficacy (*n* = 7) and low-efficacy (*n* = 7) groups. Correlation analysis between post-TTFields cell viability and cell doubling time in culture showed no significant association (R = 0.304, *P* = 0.291; Fig. 4A). Targeted next-generation sequencing (NGS) analysis identified 16 distinct gene mutations across the 14 HROC cell lines: *FGFR4*, *BRAF*, *TP53*, *PTPRJ*, *KRAS*, *MSH6*, *PIK3CA*, *APC*, *KMT2D*, *FLCN*, *MLH1*, *MSH2*, *SMAD4*, *CDKN2A*, *ERBB3*, and *NF1*. No significant differences in mutation frequency or type (nonsense, missense, insertions, deletions, or multi-hit) were observed between the two groups (Fig. 4B). Clinicopathological analysis—including patient age, gender, site of tumor origin, differentiation grade, pathological TNM stage, and molecular subtype— also revealed no substantial differences. Although microsatellite instability (MSI) status was more frequent in the high-efficacy group, the difference was not statistically significant (*P* = 0.075; Fig. 4C and Table 2).

**Table 2:** Univariate analysis of clinical and pathological characteristics for TTF sensitivity

Characteristics	High Efficacy	Low Efficacy	*P* value
*n*	7	7	
Age, mean ± sd	71.14 ± 21.67	73.29 ± 13.91	0.829
Gender, *n* (%)			0.514
female	6 (85.7%)	5 (71.4%)	
male	1 (14.3%)	2 (28.6%)	
Tumor tissue site of origin, *n* (%)			0.388
right colon	5 (71.4%)	2 (28.6%)	
transverse	1 (14.3%)	0 (0%)	
left colon	1 (14.3%)	2 (28.6%)	
liver	0 (0%)	1 (14.3%)	
sigmoid	0 (0%)	1 (14.3%)	
rectum	0 (0%)	1 (14.3%)	
Differentiation, *n* (%)			0.266
G3	6 (85.7%)	3 (42.9%)	
G2	1 (14.3%)	4 (57.1%)	
pTNM stage, *n* (%)			0.236
stage I/II	3 (42.9%)	1 (14.3%)	
stage III/IV	4 (57.1%)	6 (85.7%)	
Pathological molecular subtype, *n* (%)			0.073
spSTD	1 (14.3%)	1 (14.3%)	
spMSI	4 (57.1%)	0 (0%)	
LS	2 (28.6%)	3 (42.9%)	
CIMP-H	0 (0%)	3 (42.9%)	
MSI status, *n* (%)			0.076
MSI	7 (100%)	3 (42.9%)	
MSS	0 (0%)	4 (57.1%)	

### N-acetylated α-linked acidic dipeptidase-like 1 (*NAALADL1*) expression level as a biomarker of resistance to TTFields treatment

**Fig. 5:**
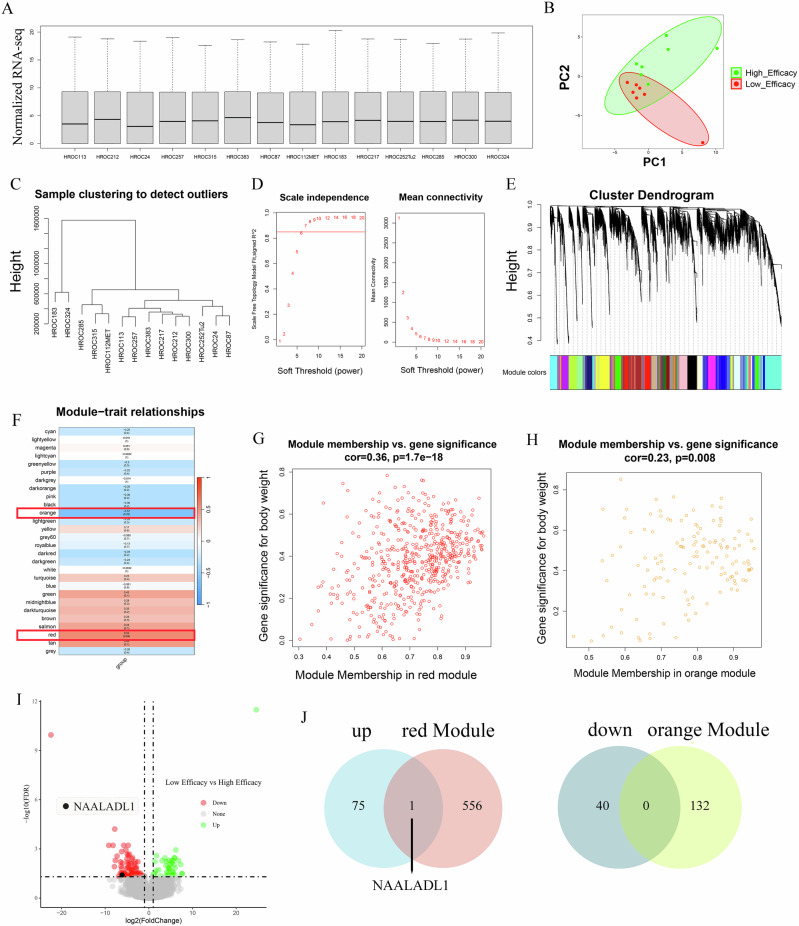
*NAALADL1* expression as a biomarker of resistance to TTFields treatment. **A** Boxplot analysis of gene expression levels across all samples confirmed successful normalization. **B** PCA revealed a clear separation between the high-efficacy and low-efficacy groups. **C** Clustering analysis did not detect extreme outliers. **D** A soft thresholding power of β = 7 with a scale-free topology fit index (R² > 0.85) was selected to construct a scale-free co-expression network. **E** A hierarchical clustering dendrogram based on the TOM identified 28 co-expression modules. **F** The red module (r = 0.56, *P* = 0.002) was positively associated with the low-efficacy group, while the orange module (r = –0.54, *P* = 0.003) was negatively associated. **G** Significant correlation of MM and GS was detected in the red module (cor = 0.36, *P* < 0.001). **H** as well as in the orange module (cor = 0.23, *P* = 0.008). **I** Differential gene expression analysis found that *NAALADL1* was significantly upregulated in the low-efficacy group. **J** Venn diagram analysis identified *NAALADL1* as the only gene overlapping between the upregulated genes in the low-efficacy group and the red module.

Subsequently, RNA-seq analysis was conducted on the 14 HROC cell lines of the high- and low-efficacy in the TTFields treatment response. As shown in Fig. 5A, the gene expression boxplot indicated successful normalization, with comparable distributions across all samples. The principal component analysis (PCA) plot (Fig. 5B) demonstrated clear separation between the high- and low-efficacy groups. To detect potential outliers, hierarchical clustering was performed based on gene expression profiles (Fig. 5C). No extreme outliers were identified, confirming the dataset’s suitability for downstream analysis. Using the weighted gene co-expression network analysis (WGCNA) package, a weighted gene co-expression network was constructed to identify modules associated with treatment efficacy. A soft-thresholding power of β = 7 was chosen to ensure scale-free topology (Fig. 5D). A hierarchical gene clustering dendrogram was generated based on the topological overlap matrix (TOM) (Fig. 5E), and modules were detected using the dynamic tree-cutting algorithm, with a minimum size of 50 genes. This process identified 28 co-expression modules. As illustrated in Fig. 5F, the module-trait heatmap showed strong correlations between treatment response and the red module (r = 0.56, *P* = 0.002) and orange module (r = –0.54, *P* = 0.003). The red module was positively associated with the low-efficacy group, while the orange module was negatively associated. Further analysis revealed significant correlations between module membership (MM) and gene significance (GS) in the red (cor = 0.36, *P* < 0.001; Fig. 5G) and orange (cor = 0.23, *P* = 0.008; Fig. 5H) modules. Volcano plot analysis (Fig. 5I) highlighted the differentially expressed genes (DEGs) between the two groups. A Venn diagram (Fig. 5J) identified *NAALADL1* as the only gene common to both the upregulated genes in the low-efficacy group and those in the red module.

### Knockdown of NAALADL1 enhances CRC sensitivity to TTFields treatment

**Fig. 6:**
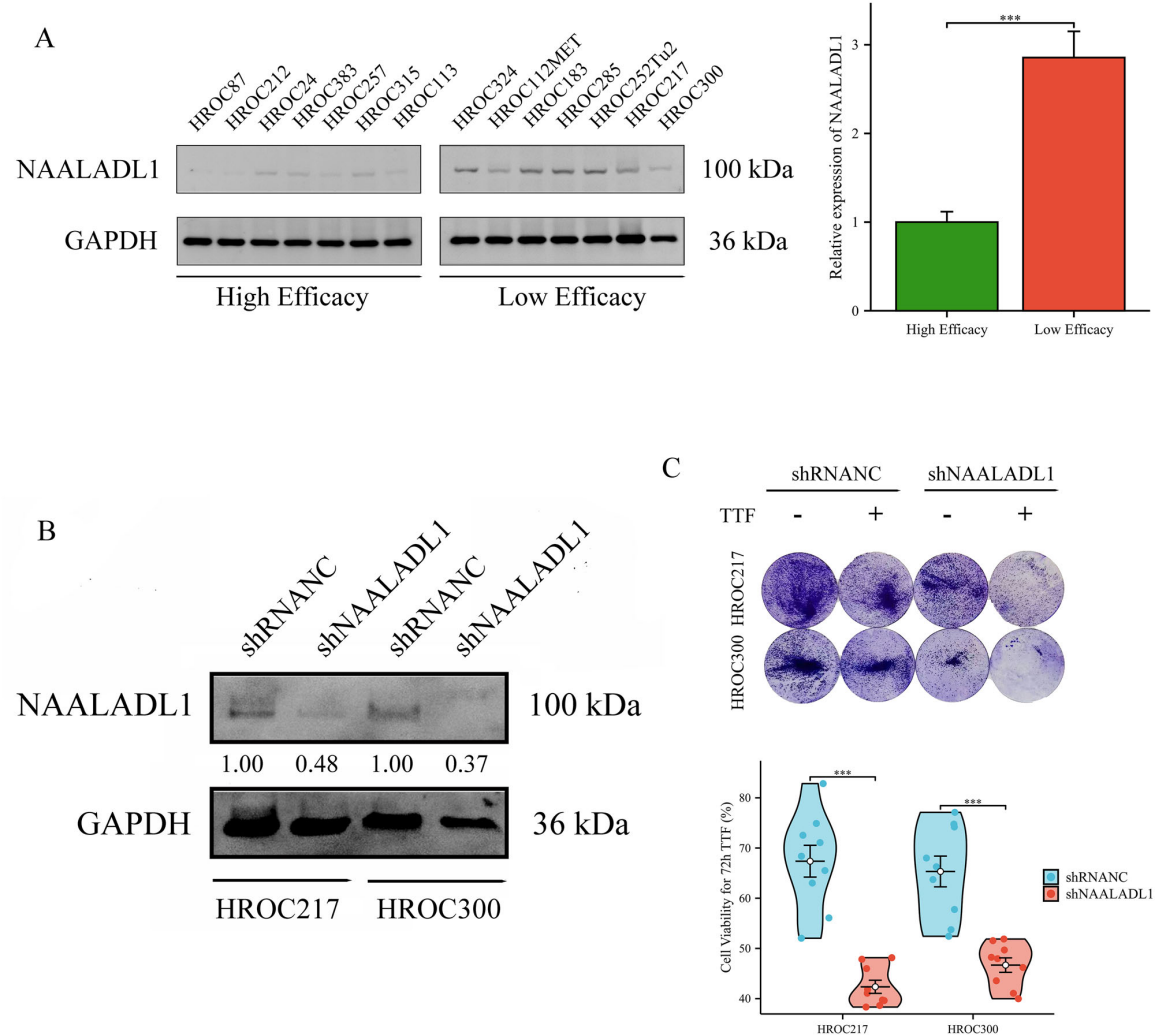
NAALADL1 knockdown enhanced CRC sensitivity to TTFields treatment. **A** WB analysis of NAALADL1 expression in 14 HROC cell lines. NAALADL1 expression in the high-efficacy group was statistically lower than in the low-efficacy group (*P* < 0.001). **B** WB analysis showed successful knockdown of NAALADL1 in HROC217 and HROC300 cells using shRNA lentiviral transduction. **C** Viability assays demonstrated that NAALADL1 knockdown enhanced sensitivity to TTFields treatment, as indicated by decreased cell viability in both HROC217 (*P* < 0.001) and HROC300 (*P* < 0.001).

Western blot (WB) results showed varying NAALADL1 protein expression across the 14 HROC cell lines, with significantly lower expression levels observed in those lines exhibiting higher sensitivity to TTFields treatment (*P* < 0.001; Fig. 6A). To functionally investigate the role of NAALADL1 in modulating the response to TTFields, shRNA knockdown experiments were performed in HROC217 and HROC300 cells, both of which originally exhibited minimal treatment efficacy (Fig. 6B). As predicted, this NAALADL1 knockdown significantly enhanced sensitivity to TTFields treatment in both cell lines, suggesting that NAALADL1 may function as a resistance-associated factor (Fig. 6C).

### Inhibition of NAALADL1 enhances CRC cell sensitivity to TTFields treatment

**Fig. 7:**
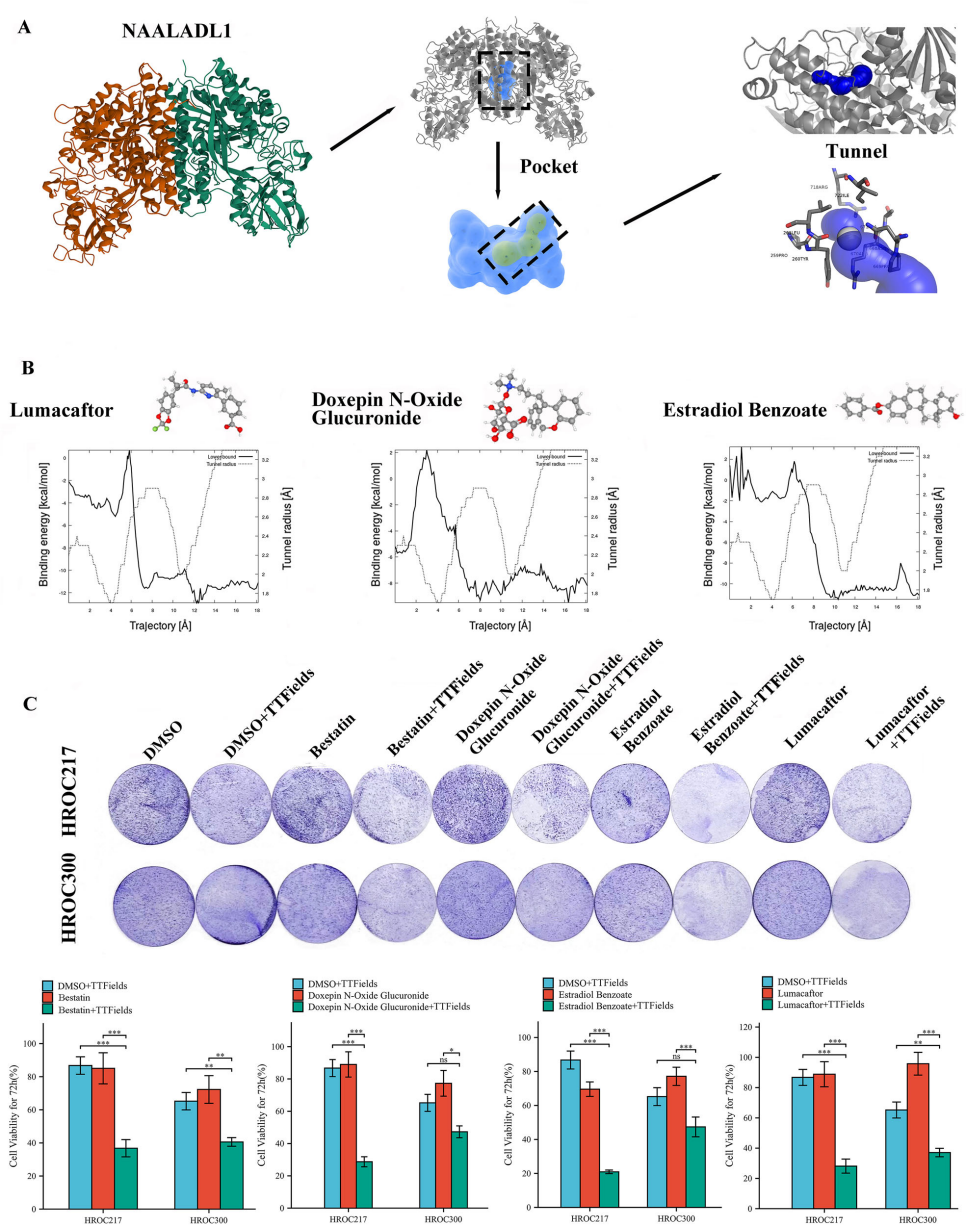
Inhibition of NAALADL1 enhances CRC sensitivity to TTFields treatment. **A** Structural analysis of NAALADL1. Left: Ribbon diagram of NAALADL1 dimer. Middle: Identification of a major binding pocket (highlighted in blue) based on geometric parameters. Right: Tunnel detection within the protein structure using CaverWeb; a key tunnel connecting the protein surface to the binding pocket is shown with a highlighted bottleneck. **B** Molecular docking results for top-ranked FDA-approved compounds—Lumacaftor, Doxepin N-Oxide Glucuronide, and Estradiol Benzoate. Each panel shows the chemical structure of the compound and the corresponding docking affinity plot, indicating potential binding strength and binding trajectory characteristics within the NAALADL1 tunnel. **C** Cell viability assays of HROC217 and HROC300 treated with DMSO (control), TTFields, NAALADL1 inhibitors alone (50 µM), or in combination with TTFields.

The structural features of NAALADL1 were analyzed using CaverWeb 2.0 (https://loschmidt.chemi.muni.cz/caverweb/)^23^, focusing on its binding pockets and tunnels. Among the identified pockets, the top-ranked one was selected for further analysis. This pocket contains key catalytic residues (A:L227, B:L227, A:E394, B:E394, A:D623, B:D623) and achieved a relevance score of 100%, with a calculated volume of 3462 Å³ and a druggability score of 0.153. The corresponding tunnel features a bottleneck radius of 1.6 Å, a total length of 24.1 Å, and a distance of 19.1 Å from the protein surface. It also displayed a curvature of 1.3 and a throughput value of 0.62, indicating potential for ligand accessibility. The tunnel consists of 34 residues and contains a single bottleneck, which may influence molecular transport and interaction dynamics within the protein (Fig. 7A and Supplementary Data 2). Molecular docking analysis identified 12 FDA-approved compounds with strong binding affinities to the NAALADL1 channel, exhibiting binding energies between –10.6 and –10.0 kcal/mol (Table 3). The top three candidates—Doxepin N-Oxide Glucuronide (ZINC000095618886), Estradiol Benzoate (ZINC000003881345), and Lumacaftor (ZINC000064033452)—were selected based on binding energy (Fig. 7B), along with Bestatin, a known NAALADL1 inhibitor^24^. All four compounds showed IC₅₀ values above 50 µM in both cell lines (Supplementary Fig. 2). Cell viability assays were then conducted to assess synergistic effects of Bestatin, Doxepin N-Oxide Glucuronide, Estradiol Benzoate, and Lumacaftor in combination with TTFields on HROC217 and HROC300 cells. Of the drugs tested, Lumacaftor and Bestatin demonstrated statistically significant synergistic effects in both HROC cell lines (Fig. 7C).

**Table 3:** Top 12 FDA-approved compounds with high binding affinities to the NAALADL1 protein channel identified by molecular docking

ZINC ID	binding energy (kcal/mol)
ZINC000064033452	–10.6
ZINC000003881345	–10.4
ZINC000095618886	–10.3
ZINC000116473771	–10.3
ZINC000000897408	–10.2
ZINC000003779720	–10.2
ZINC000031425361	–10.2
ZINC000089224316	–10.2
ZINC000000577115	–10.1
ZINC000003821234	–10.1
ZINC000012494489	–10.1
ZINC000001539579	–10

### Downregulation of NAALADL1 induces G2/M arrest via microtubule stabilization in CRC cells

**Fig. 8:**
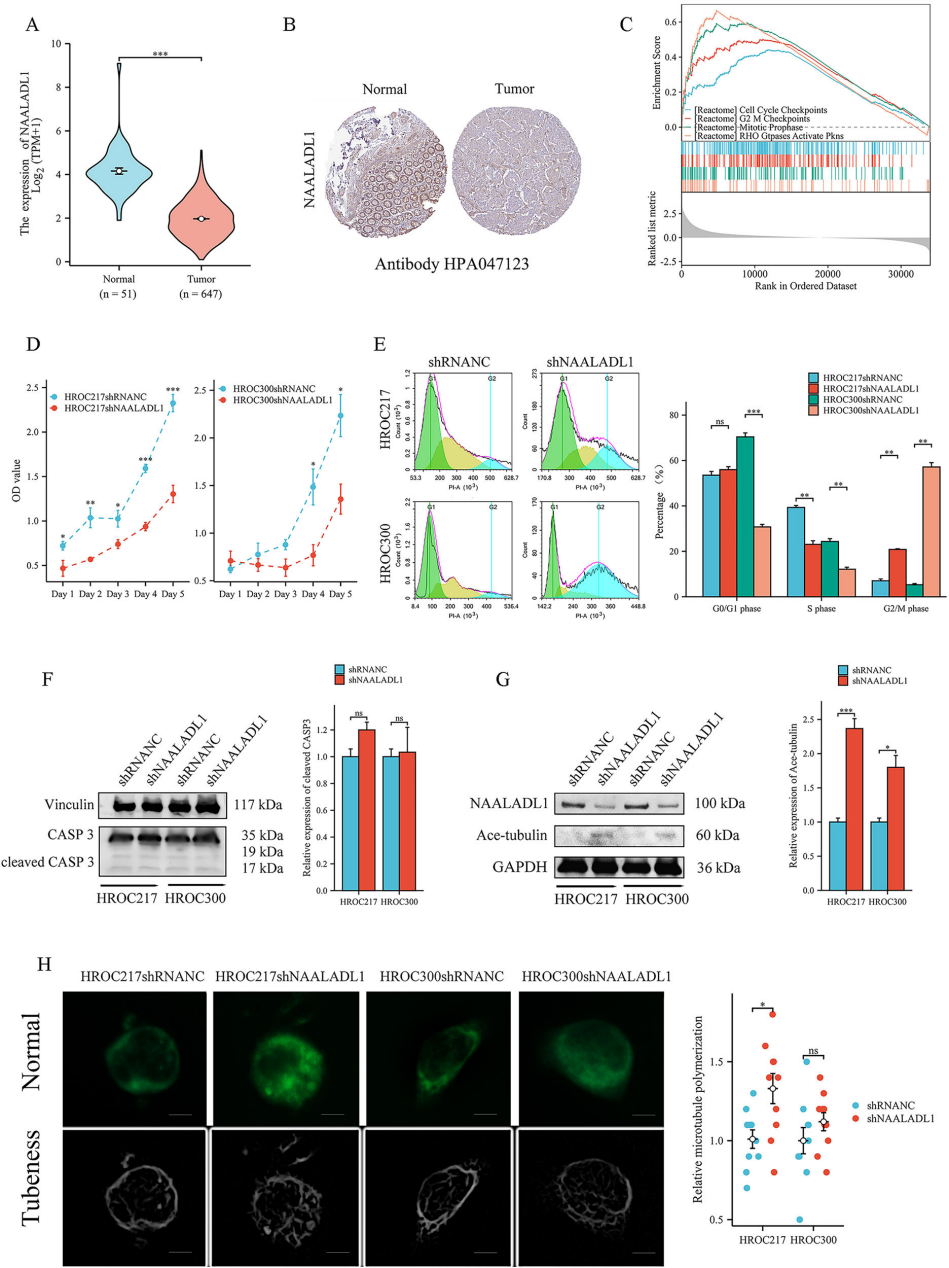
Downregulation of NAALADL1 induces G2/M phase arrest via enhanced microtubule stabilization in CRC cells. **A** NAALADL1 was significantly downregulated in tumor tissues compared with normal tissues based on dataset analysis from public databases (*P* < 0.001). **B** Representative immunohistochemical staining demonstrated reduced NAALADL1 protein expression in CRC tissues compared to normal tissues. **C** GSEA revealed NAALADL1 was significantly enriched in the Reactome pathways Mitotic Prophase, Cell Cycle Checkpoints, G2/M Checkpoints, and Rho GTPases Activate PKNs. **D** Crystal violet assays showed that NAALADL1 knockdown significantly suppressed the proliferation of HROC217 and HROC300. **E** FC analysis revealed a significant increase in the proportion of G2/M phase cells upon NAALADL1 knockdown in both HROC217 (*P* < 0.01) and HROC300 (*P* < 0.01). **F** WB analysis of cleaved caspase-3 expression in NC and NAALADL1 knockdown cell lines revealed no significant difference. **G** WB analysis demonstrated that NAALADL1 knockdown upregulated Ace-tubulin levels in HROC217 (*P* < 0.001) and HROC300 (*P* < 0.05). **H** Immunofluorescence staining showed significantly increased microtubule polymerization in HROC217 cells following NAALADL1 knockdown (*P* < 0.05). Scale bar = 5 µm.

To further investigate the specific role of NAALADL1 in CRC, we analyzed its expression and potential functions using public databases, including The Cancer Genome Atlas (TCGA) and the Human Protein Atlas. Public data analyses revealed significantly lower *NAALADL1* expression in tumor tissues compared to normal tissues (Fig. 8A), a finding confirmed at the protein level by immunohistochemical staining (Fig. 8B). Gene set enrichment analysis (GSEA) showed significant enrichment of *NAALADL1* in cell cycle- and mitosis-related pathways, including the Reactome pathways Mitotic Prophase, Cell Cycle Checkpoints, G2/M Checkpoints, and Rho GTPases Activate PKNs (Fig. 8C). Functional assays demonstrated that NAALADL1 knockdown significantly reduced proliferation in HROC217 and HROC300 cells (Fig. 8D). FC further revealed a marked increase in the G2/M-phase cell population following NAALADL1 knockdown (Fig. 8E). WB analysis showed no significant changes in cleaved caspase-3 levels (Fig. 8F). Notably, NAALADL1 silencing strongly increased acetylated tubulin expression, suggesting enhanced microtubule stability (Fig. 8G). This was further supported by immunofluorescence staining, which revealed significantly increased microtubule polymerization in NAALADL1-knockdown HROC217 cells compared to controls (Fig. 8H).

## Discussion

TTFields is a novel, non-invasive physical therapy that applies intermediate-frequency, low-intensity alternating electric fields to disrupt cancer cell division^25–29^. Its clinical potential was first demonstrated in GBM. The pivotal Phase III EF-14 trial demonstrated significantly improved PFS and OS when TTFields was combined with temozolomide compared to standard chemotherapy^30^. After FDA approval in 2004 for recurrent GBM, TTFields’ application has expanded to other solid tumors. The STELLAR trial^31^ reported a median OS of 18.2 months in malignant pleural mesothelioma, while the ongoing LUNAR trial is assessing efficacy in NSCLC^12^. In vitro and early-phase studies are also investigating its use in pancreatic and ovarian cancers^32–34^.

Although TTFields is gaining interest for solid tumors, its application in CRC remains under investigation. The first in vitro study showing TTFields-induced growth inhibition in CRC cell lines was published 2019^35^, highlighting its antiproliferative effects and potential to enhance sensitivity to 5-fluorouracil.In advanced rectal cancer, especially stage III low-position tumors, neoadjuvant therapies like radiation are often needed to secure clear surgical margins^36–40^. However, radiation can cause pelvic floor edema and scarring, complicating surgery and causing other adverse effects^41–45^. Given its minimal effect on normal tissue, TTFields may serve as a promising adjunct and potentially help overcome therapeutic resistance and enhance the efficacy of existing treatment regimens.

In this study, we systematically interrogated TTFields sensitivity across a panel of 21 low-passage CRC cell lines and identified substantial intertumoral heterogeneity, indicating that intrinsic molecular features govern treatment responsiveness.

Ultrastructural examination by STEM revealed substantial morphological changes in TTFields-sensitive cells, including pseudopodia loss, chromatin condensation, mitochondrial swelling, and cytoplasmic vacuolization—morphological features consistent with cellular stress and apoptotic progression^46,47^. These findings suggest that TTFields may disrupt cellular integrity, triggering molecular events leading to cell death. Proteomic analysis of TTFields-sensitive cell lines showed extensive changes in protein expression across several key pathways, primarily linked to DNA damage and metabolic dysregulation, indicating that TTFields impairs multiple essential cellular functions. Immunofluorescence staining confirmed microtubule depolymerization, nuclear deformation, and mitotic arrest after TTFields treatment, further supporting the hypothesis that TTFields acts through mitotic disruption. FC analysis showed a marked increase in apoptotic cells post-TTFields, especially in highly responsive lines. Taken together, the results indicate that TTFields is associated with apoptosis potentially driven by cytoskeletal disruption, metabolic imbalance, and genomic instability. A better understanding of these mechanisms may help clarify its therapeutic relevance in CRC and guide the development of rational combination strategies to improve clinical outcomes.

Mutation analysis identified 16 gene mutations across the 14 HROC cell lines, including alterations in KRAS, TP53, and BRAF, among others. Nevertheless, no substantial differences in mutation type or frequency were found between high- and low-response groups, suggesting that TTFields efficacy is not dependent on a specific oncogenic driver. Similarly, clinicopathological comparisons showed no significant differences in age, gender, tumor location, histological differentiation, or TNM stage. Although the high-response group showed a greater proportion of MSI cases, the trend was not statistically significant, possibly due to the study’s limited sample size. Collectively, these results indicate that TTFields response in CRC is governed by complex, multifactorial mechanisms that are not predictable by proliferation, mutation status, or standard clinical features.

To explore the molecular basis of differential TTFields response, transcriptomic analyses were performed to identify candidate resistance-associated genes. Among differentially expressed genes, *NAALADL1* emerged as the only gene both upregulated in low-response lines and enriched in the resistance module, suggesting a potential role in TTFields resistance. WB confirmed elevated NAALADL1 protein in TTFields-resistant lines. Functional validation via shRNA-mediated knockdown of NAALADL1 in resistant CRC lines significantly reduced viability after TTFields treatment, indicating increased sensitivity and supporting its role in modulating resistance.

Given that NAALADL1 is an enzyme, and enzymes represent attractive pharmacological targets due to their defined catalytic sites and druggable structural features^48,49^, we further examined its structural properties. Computational structural analyses identified several putative binding pockets and channels, including sites containing predicted catalytic residues. These findings provide a rationale for pharmacological targeting of NAALADL1.

Virtual screening of FDA-approved drugs identified several compounds with strong binding to NAALADL1. Lumacaftor and Bestatin, in particular, significantly enhanced TTFields efficacy in the most resistant CRC cell lines. This suggests that combination therapy may help overcome resistance and improve treatment outcomes. Given that both knockdown and inhibition of NAALADL1 increased TTFields sensitivity in resistant CRC cell lines, its function and role in modulating TTFields response warranted further investigation. NAALADL1, a close homolog of glutamate carboxypeptidase II—a zinc-dependent metallopeptidase extensively studied as a therapeutic and diagnostic target in cancer and neuro pathologies^50–54^ —remains functionally poorly understood. In this study, bioinformatic and immunohistochemical analyses showed consistently lower NAALADL1 expression in CRC compared to normal tissues across multiple public datasets. GSEA linked NAALADL1 to pathways regulating the cell cycle, including G2/M checkpoint control, mitotic prophase, and cytoskeletal remodeling via Rho GTPases. These findings point to a role for NAALADL1 in cell cycle progression and microtubule dynamics.

To further investigate its biological function, NAALADL1 was knocked down via shRNA in TTFields-resistant CRC cell lines. This markedly suppressed proliferation and induced G2/M cell cycle arrest, as confirmed by FC. Cleaved caspase-3 levels remained unchanged, indicating that growth inhibition resulted from cell cycle blockade rather than apoptosis. Mechanistically, knockdown of NAALADL1 increased Ace-tubulin levels, a well-established marker of microtubule stabilization^55–57^. We therefore propose that NAALADL1 inhibition perturbs microtubule dynamics, leading to G2/M arrest and increasing cellular vulnerability to TTFields, which also interferes with mitotic processes. Further investigation of additional G2/M regulators, apoptosis markers, and survival pathways will be required to fully delineate this mechanism.

**Fig. 9:**
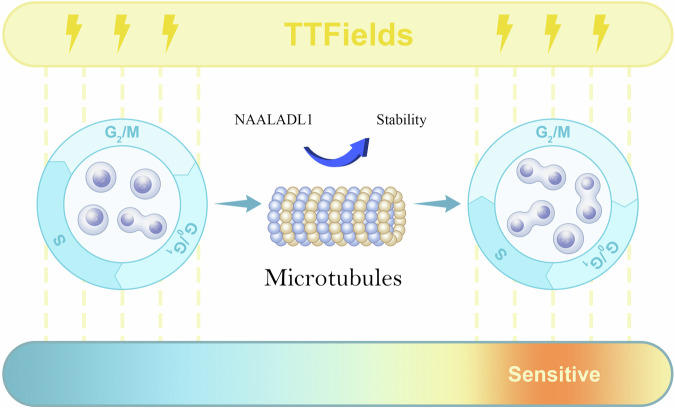
Proposed role of NAALADL1 in TTFields response regulation in CRC. NAALADL1 inhibition potentiates TTFields by stabilizing microtubules, inducing spindle defects and G2/M arrest - thereby increasing the vulnerable cell population susceptible to TTFields’ mitotic interference.

Proper mitosis requires dynamic microtubule remodeling for spindle assembly and chromosome segregation^58^. Both excessive stabilization and destabilization disrupt this balance^59,60^, impairing spindle formation and accurate chromosome alignment. Cells may activate the spindle assembly checkpoint, leading to G2/M cell cycle arrest^61,62^, which preserves genomic integrity but increases susceptibility to mitosis-targeting agents. Based on these findings, we propose that in TTFields-resistant CRC cells, NAALADL1 knockdown or inhibition enhances microtubule stability, disrupting their dynamic remodeling and impairing spindle formation. This leads to G2/M arrest. Since TTFields interferes with microtubule dynamics during mitosis, a higher proportion of cells arrested at G2/M may potentiate its effects (Fig. 9).

This study has several limitations. Although low-passage CRC cell lines derived from patient tumors and PDX models retain important clinical features, our experiments were mainly conducted in two-dimensional in vitro systems, as current our TTFields exposure devices are technically limited in accommodating three-dimensional organoid systems or in vivo PDX models. In addition, while our data suggest that *NAALADL1* suppression enhances TTFields sensitivity through microtubule stabilization and G2/M arrest, the precise molecular mechanisms remain to be further elucidated.

In conclusion, we demonstrate that TTFields efficacy in CRC is governed by intrinsic molecular heterogeneity and identify NAALADL1 as a functional modulator of resistance. By linking microtubule regulation to TTFields susceptibility, this study provides a mechanistic rationale for biomarker-driven patient stratification and rational combination strategies in colorectal cancer.

## Methods

### HROC cell lines

Twenty-one low-passage CRC cell lines established from PDX models or directly from primary/metastatic CRC tissues from the HROC collection were selected. Cells were cultured in DMEM-F12 medium with 2 mM L-glutamine and 10% FCS at 37 °C and 5% CO_2_, with medium changes when necessary and passaging at 70–80% confluence. All cell lines were authenticated by STR profiling and tested for mycoplasma. Detailed cell line information is listed in Supplementary Table 1.

### Patient characteristics and pathological assessment

Demographic and clinical data, including age, gender, tumor site, histological grade, and tumor stage, were recovered from medical records of the 21 CRC patients that gave the origin of the HROC cell models used in this study. Molecular classification included sporadic standard chromosomal instable, sporadic MSI, Lynch syndrome, and CpG island methylator phenotype high status. MSI was evaluated using the Bethesda marker panel (BAT25, BAT26, D2S123, D5S346, D17S250). All pathological evaluations were performed by the Institute of Pathology, Rostock University Medical Centre. Comprehensive data were summarized in Supplementary Table 1.

### TTFields treatment and cell viability assay

CRC cells were seeded on prewetted coverslips and cultured in 6-well plates with 2 × 10⁶ cells/well. The following day, coverslips were transferred into an inovitro dish and left untreated (living control) or treated with TTFields using the NovoTTFields-100A™ System (Novocure Ltd., Haifa, Israel) under the following conditions: Coverslips were placed in ceramic culture dishes positioned between two pairs of insulated electrodes to generate alternating electric fields in orthogonal directions. Field direction alternated automatically to ensure uniform exposure. Temperature and impedance were continuously monitored to maintain stable field delivery during experiments. TTFields were applied at a frequency of 100 kHz with an electric field intensity of 1-3 V/cm continuously for 72 hours (Supplementary Fig. 1). Cell viability was assessed using the 0.2% crystal violet assay. After staining, the dye was solubilized in 1% SDS and absorbance measured at 570 nm. Cell viability (%) = (Absorbance of treated cells − Absorbance of blank) / (Absorbance of living control cells − Absorbance of blank) × 100.

### STEM analysis

CRC cells were seeded onto sterile coverslips and divided into two groups: the control group, without TTFields treatment, and the TTFields-treatment group, which was exposed to TTFields (100 kHz, 1-3 V/cm) for 48 h. After treatment, the cells were detached from the coverslips and were fixed in 2.5% glutaraldehyde, embedded in low-melt agarose, and post-fixed with 1% osmium tetroxide. Samples were dehydrated, infiltrated with Epon resin, and polymerized. Ultrathin sections (50–70 nm) were stained with uranyl acetate and lead citrate, prior carbon-coating (3 nm). Finally, the morphological characteristics of the cells were observed and imaged in a field emission scanning electron microscope (Zeiss Merlin VP compact, Carl Zeiss Oberkochen, Germany) using the STEM mode with dedicated combined dark field and bright field detection at an acceleration voltage of 15 kV and a working distance of approximately 2.7 mm.

### Doubling time analysis

Cells were seeded into 24 well plates (10⁴–10⁵ cells / mL) and counted at 0, 24, 48, and 72 h. Growth curves were plotted and doubling times (Td) calculated using the standard logarithmic formula: Td = (t × log2) / (log(N_t_) – log(N_0_)), where t is the time interval in hours, N_t_ is the cell count at the given time point, and N_0_ is the initial cell count.

### NGS analysis

Genomic DNA was extracted from cultured HROC cell lines utilizing the Precellys Tissue DNA Kit (PEQLAB Life Science, Erlangen, Germany), following the manufacturer’s protocol. DNA quality control measures mandated a minimum concentration of 25 ng/μL, and integrity was assessed using the DNA integrity number, with a threshold set at ≥ 5. High-throughput NGS was subsequently carried out by Centogene AG (Rostock, Germany) using a clinically validated solid tumor gene panel optimized for oncogenic profiling, targeting the full coding sequences of 105 well-characterized cancer-associated genes alongside hotspot regions from an additional 146 oncogenes and tumor suppressor genes^37^.

### RNA sequencing

Total RNA was extracted from CRC cell lines using the RNeasy Plus Mini Kit (Qiagen, Hilden, Germany) according to the manufacturer’s instructions. RNA quality was then assessed using a Bioanalyzer 2100 (Agilent Technologies, Santa Clara, CA, USA), and RNA integrity was determined by the RNA Integrity Number, with a value ≥ 7.0 considered suitable for downstream library preparation and sequencing. The constructed cDNA libraries were quantified using a Qubit 4 Fluorometer (Thermo Fisher Scientific, Waltham, MA, USA) and analyzed for fragment size distribution on an Agilent 2100 Bioanalyzer. Qualified libraries were sequenced on the Illumina NovaSeq 6000 platform using a 150 bp strategy, generating approximately 50 million reads per sample. FastQC (v0.11.9) was used to assess raw data quality, and Trimmomatic (v0.39) was applied to remove low-quality reads and adapter sequences. For alignment, HISAT2 (v2.2.1) was used to map clean reads to the reference genome (GRCh38). Transcriptome assembly and quantification were performed using StringTie (v2.1.7), and gene expression levels were calculated with featureCounts (v2.0.1). The original transcriptomic data from this study have been uploaded to the National Center for Biotechnology Information Gene Expression Omnibus database (Supplementary Table 2).

### MS analysis

Proteins from cell pellets were processed using the Mini MS EasyPep Kit (Thermo Fisher Scientific). Briefly, cell pellets were resuspended in lysis buffer, and their protein concentration was determined by BCA assay. Equal amounts of protein were reduced with DTT, alkylated with IAA, and subsequently subjected to enzymatic digestion with trypsin to generate peptides. Peptides were desalted using C18 solid-phase extraction. Two hundred ng of the purified peptides were loaded onto C18 EvoTip disposable trap columns (EV1137, 15 cm×150 µm, 1.5 µm) and chromatographically separated using the extended 15 SPD method (88 min gradient, 220 nl/min) with an EvoSep One (EvoSep, Denmark). Subsequently, peptides were measured using the timsTOF fleX mass spectrometer (Bruker, Germany) in a label-free data-independent acquisition mode.

### Lentiviral transduction

To determine puromycin selection pressure, 2 × 10⁴ HROC217 and HROC300 cells were seeded in wells of 96-well plates and treated with a concentration gradient of puromycin (0–3.0 μg/mL). After 72 hours, viability was assessed via crystal violet staining, and the lowest lethal dose for untransduced cells was selected (Supplementary Fig. 3). Commercially available lentiviral particles targeting NAALADL1 (Catalog No. TL311278V, OriGene Technologies GmbH, Herford, Germany; A-D mix in equal volumes) and corresponding control vectors (Catalog No. TR30021V, OriGene Technologies GmbH), which contain random sequences of the same length as the shRNA but without human targets, were used to infect the HROC217 and HROC300 cell lines (HROC217shRNANC, HROC217shNAALADL1, HROC300shRNANC, and HROC300shNAALADL1). Cells were seeded (1 × 10⁵ cells / well, 48-well plate), incubated overnight, and infected in the presence of 6 μg/mL polybrene (Merck, Darmstadt, Germany) at a multiplicity of infection = 50. After 24 h, medium was changed and cells were cultured for an additional 48 h. Transduction efficiency was monitored by GFP fluorescence, and puromycin selection was applied when necessary.

### WB assay

A total of 1 × 10⁶ cells were lysed in 500 µL RIPA buffer (Thermo Fisher Scientific) with protease inhibitor (Roche, Basel, Switzerland). SDS-PAGE was performed using 10% polyacrylamide gel (TGX™ FastCast™, Bio-Rad, Munich, Germany) at 100 V for 1–1.5 h. Proteins were transferred to a nitrocellulose membrane using Bio-Rad’s semi-dry transfer system (Trans-Blot Turbo) at 1 A for 30 min. Membranes were blocked with 5% skim milk for 1 h, followed by overnight incubation at 4 °C with diluted primary antibodies. The next day, membranes were washed with TBST, incubated with secondary antibody for 1 h at room temperature. Detection was performed using a fluorescent imaging system, and band intensity was analyzed to quantify target protein expression. GAPDH and Vinculin were used as internal controls. Antibody details and dilutions are listed in Supplementary Table 3.

### Cellular functional assays

For cell proliferation, log-phase cells were seeded in 96-well plates at 1–2 × 10⁴ cells/well in 100 µL culture medium and incubated at 37 °C with 5% CO_2_ for 1 to 5 days. Cells were stained with 0.2% crystal violet for 15 min at room temperature, washed with distilled water, and air-dried. Stained wells were imaged, and 100 µL of 1% SDS was added to solubilize the dye. Absorbance at 570 nm was measured using a microplate reader. OD values were used to generate cell growth curves. For cell cycle analysis, log-phase cells were harvested, and fixed in 70% methanol at 4 °C for 30 min. Cells were centrifuged again, washed with DPBS, and resuspended in 500 µL DPBS. RNase A (100 µg/mL) treatment was performed at 37 °C for 30 min, followed by staining with 50 µg/mL PI in the dark for 30 min. DNA content was analyzed by FC (NovoCyte Quanteon, Agilent, San Diego, USA) to determine cell cycle distribution (G0/G1, S, G2/M). For non-transfected cells, log-phase cells (1 × 10⁶) were washed with DPBS and resuspended in 500 µL Annexin V binding buffer. Cells were stained with 10 µL Annexin V-APC and 10 µL PI per kit instructions, incubated in the dark for 15 min at room temperature, and analyzed by FC for apoptosis analysis. For transfected cells, cleaved Caspase-3 was detected by WB to avoid GFP interference.

### Immunofluorescence

Cells were either treated or not treated with TTFields to assess its effects on microtubule structures. Fixation was performed using pre-chilled 70% methanol (–20 °C) for 30 min. Immunofluorescence staining for tubulin was conducted using Tubulin Tracker™ Green kit (Thermo Fisher Scientific), with incubation at 4 °C for 2 h. Nuclear staining was performed using DAPI (1:5000). Fluorescence microscopy (Zeiss Axio Observer 7 with axiocam 506 mono and the ZenPro software, version 2.3) was used to analyze the effects of TTFields treatment on the microtubule structure.

Tubulin Tracker™ Green kit was also used for staining polymerized microtubules. Quantitative analysis of microtubule polymerization was conducted using the Tubeness plugin in Fiji. Raw images were initially converted to 8-bit grayscale to standardize processing. Tubular structures were subsequently enhanced via Tubeness filtering (σ = 0.1) to highlight microtubule-like features. After image enhancement, the “Mean Intensity” analysis was performed to quantify the average gray value of polymerized microtubule signals within each cell, serving as a relative measure of microtubule polymerization levels.

### Protein tunnel prediction and virtual screening

To investigate potential binding sites and channels of the NAALADL1 protein, its 3D structure (PDB ID: 4TWE) was obtained from the Protein Data Bank (https://www.rcsb.org/). The structure was analyzed using CaverWeb 2.0 (https://loschmidt.chemi.muni.cz/caverweb/, accessed on 2024.10.1)^38^, which employs the Fpocket algorithm for pocket and tunnel detection. After identifying these structural features, virtual screening was performed using an FDA-approved drug library from the ZINC database (https://zinc.docking.org/, accessed on 2024.10.1). Molecular docking with AutoDock Vina evaluated binding affinities of library compounds with the identified channels. Binding energies and interaction maps were used to rank compounds and select top candidates for further analysis and validation.

### Drug sensitivity and TTFields combination assay

Logarithmic phase cells were seeded in 96-well plates at a density of 2 × 10⁴ cells per well in 100 μL of medium. After 24 h, the cells were treated with drugs—Bestatin, Doxepin N-Oxide Glucuronide, Estradiol Benzoate, and Lumacaftor—at gradient concentrations of 50 μM, 25 μM, 12.5 μM, 6.25 μM, 3.13 μM, 1.56 μM, and 0.78 μM, respectively. The living control was complete medium containing 0.5% DMSO. The plates were then incubated for 72 h, followed by crystal violet staining as described before. The IC_50_ values were calculated using Prism 9.0 software. To evaluate the combined effects of TTFields and drug treatments, four experimental groups were established: (1) NC group: cells treated with 0.5% DMSO without TTFields exposure for 72 h. (2) Drug alone group: cells treated with the listed drugs at the respective concentrations based on the drug sensitivity assay for 72 h. (3) TTFields alone Group: cells exposed to TTFields while being maintained in a 0.5% DMSO-containing medium for 72 h. (4) Combination Group: cells treated with the respective drugs while simultaneously being exposed to TTFields for 72 h.

### Bioinformatics analysis

The expression of the *NAALADL1* gene in colon adenocarcinoma was analyzed using R-based bioinformatics tool Xiantao Academic (https://www.xiantaozi.com/, accessed on 2024.10.1). Whole transcriptome sequencing data from 647 carcinoma and 51 normal tissues were obtained from TCGA databases (https://portal.gdc.cancer.gov/, accessed on 2024.10.1). Unpaired analyses were performed to assess NAALADL1 mRNA expression. Additionally, immunohistochemical data from the Human Protein Atlas (https://www.proteinatlas.org/, accessed on 2024.10.1) were used to compare expression in CRC versus normal colonic mucosa. To explore NAALADL1 function in CRC, GSEA was performed on the transcriptomic data using the R package clusterProfiler (v4.4.4) with the MSigDB c2.cp.all.v2022.1.Hs.symbols.gmt gene set (via msigdbr), identifying significantly enriched pathways associated with high and low NAALADL1 expression.

RNA-seq data were normalized using DESeq2 (v1.32.0). Weighted Gene Co-expression Network Analysis (WGCNA, v1.70-3) was applied to construct gene co-expression networks and identify functional modules. Prior to network construction, PCA was used to assess global patterns and detect batch effects; hierarchical clustering removed outliers. Pearson correlation was calculated between gene pairs, and a soft threshold of β = 7 was applied to ensure scale-free topology. The expression matrix was transformed into an adjacency matrix and then into a TOM, followed by average linkage hierarchical clustering. Modules were identified using the dynamic tree-cutting algorithm (minModuleSize = 50) and merged using a cut height of 0.25 and deep split = 3. Modules significantly associated with TTFields sensitivity (*P* < 0.05) were further analyzed. Differential expression analysis between high- and low-efficacy TTFields groups was performed with limma, identifying DEGs using the same criteria (adj.P.Val < 0.05, |log2FC | > 1). Upregulated genes in the low-efficacy group were intersected with resistance modules, and those in the high-efficacy group with sensitivity modules, yielding candidate biomarkers and therapeutic targets. To evaluate the impact of TTFields treatment, MS was used to profile proteomic changes after 48 h. Differential expression analysis using limma identified significantly altered proteins ( | log2FC | > 1, adj.P.Val < 0.05), which were visualized in volcano plots and analyzed for GSEA enrichment.

### Study approval

The study was conducted according to the guidelines of the Declaration of Helsinki. All patients gave informed written consent to participate in the study and all procedures were approved by the Ethics Committee of the University of Rostock University Medical Centre (Reference numbers: II HV 43/2004 and A 45/2007) in accordance with generally accepted guidelines for the use of human material.

### Statistical analysis

All statistical analyses were performed with the SPSS 19.0 and R 4.0.3 software programs. Quantitative data were summarized using mean and standard deviation. *t*-tests and ANOVA were used for analysis. Categorical data were described with frequencies and percentages and the Chi-Square test and Fisher’s exact test were used. A two-sided *P* < 0.05 was considered significant. *P < 0.05; **P < 0.01; ***P < 0.001.

## Supplementary information

Supplementary information

Supplementary Data 1

Supplementary Data 2

## Data Availability

The data supporting the findings from this study are available within the manuscript and its supplementary information. The raw RNA-seq data have been deposited at NCBI sequence read archive (SRA) database with accession numbers listed in Supplementary Table 2. The *NAALADL1* expression data in human CRC tissue analyzed in this study were obtained from The Cancer Genome Atlas (TCGA), publicly available at the Genomic Data Commons (GDC) Data Portal (https://portal.gdc.cancer.gov/, accessed on 2024.10.1). Immunohistochemical data of NAALADL1 expression were obtained from the Human Protein Atlas (HPA; https://www.proteinatlas.org/, accessed on 2024.10.1).
