# Supplementary material for: NAALADL1 modulates cellular resistance to Tumor Treating Fields in colorectal cancer
Source: NPJ Precis Oncol. 2026 May 26;10:191. doi: 10.1038/s41698-026-01492-0 (PMC13213047; doi:10.1038/s41698-026-01492-0)
Supplement: Supplementary file 1 — Supplementary information [file 41698_2026_1492_MOESM1_ESM.pdf]

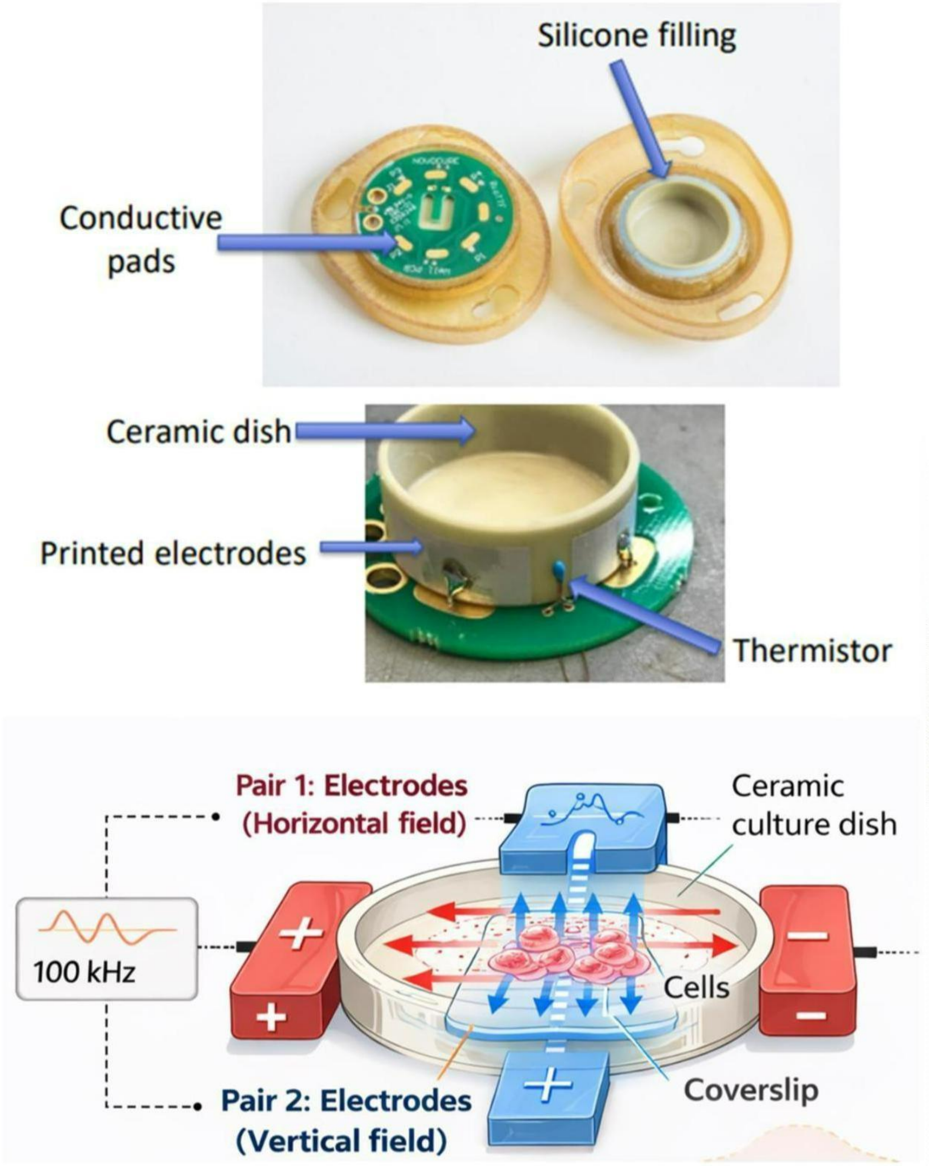

Supplementary Figure 1. TTFields in vitro exposure system and treatment parameters.

Supplementary Data 1. Raw data from MS-based proteomics.

Supplementary Data 2. Detailed data on NAALADL1 binding pocket and tunnel.

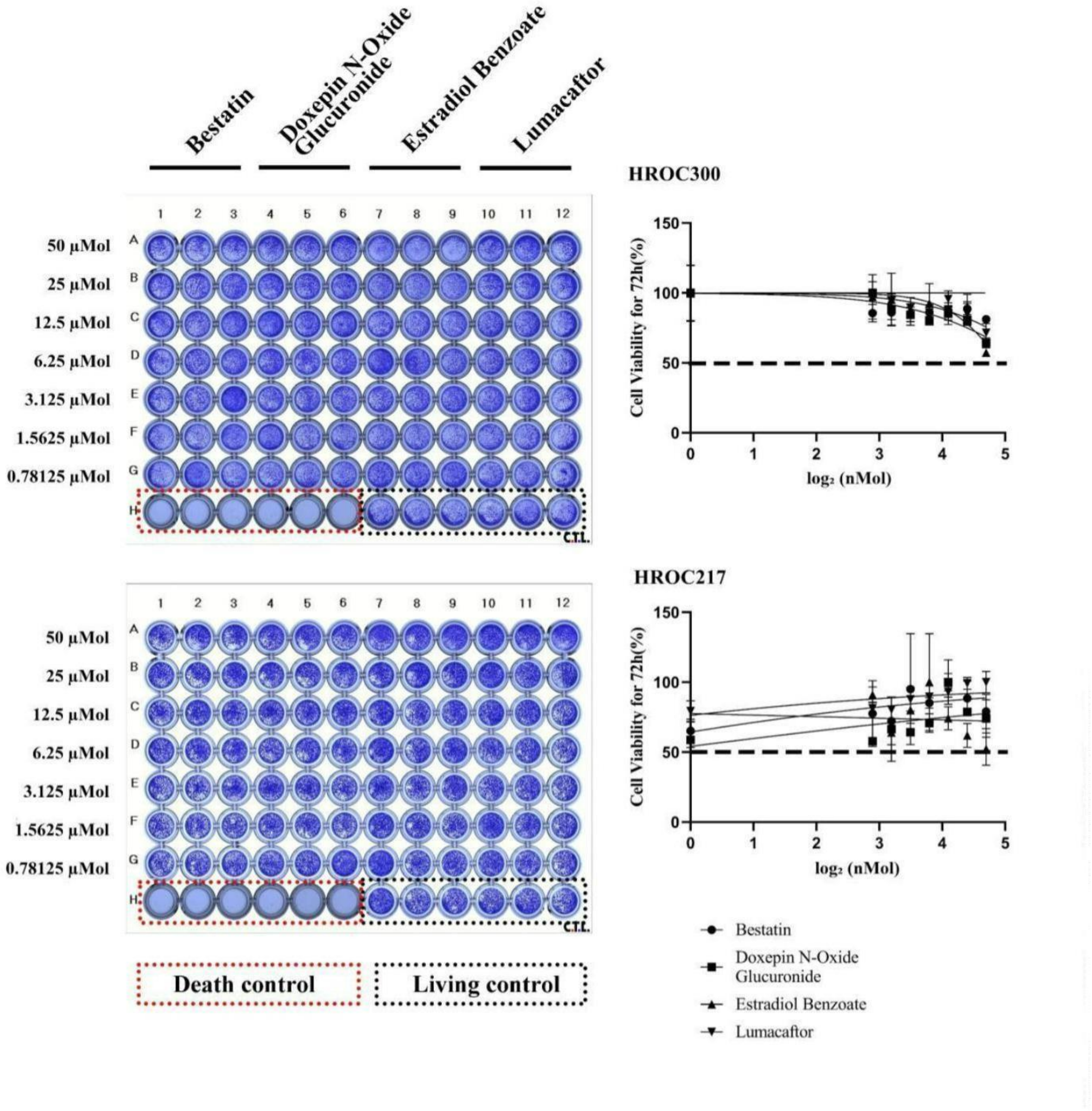

Supplementary Figure 2. IC_{50} values of Bestatin, Doxepin N-Oxide Glucuronide, Estradiol Benzoate, and Lumacaftor for HROC300 and HROC217.

ID	Cell line full name in the biobank	Sample source	Cell viability after TTF for 72 hour % (Mean ± SD)	Patient age	Gender	Tumor location	G grading	Pathological staging	MSI status	Molecular subtype	Cell Doubling Time (h)
HROC87	HROC87 T0 M2	PDX	23.8 ± 3.58	76	Female	Right colon	3	2	MSI	spSTD	35
HROC212	HROC212	Patient	26.9 ± 6.57	74	Female	Right colon	3	4	MSI	spMSI	28
HROC24	HROC24 T1 M1	PDX	28.02 ± 6.04	98	Male	Right colon	2	1	MSI	spMSI	26
HROC383	HROC383	Patient	30.41 ± 8.18	83	Female	Transverse	3	2	MSI	spMSI	49
HROC257	HROC257	Patient	32.29 ± 7.92	84	Female	Right colon	3	3	MSI	spMSI	62
HROC315	HROC315 T1 M2	PDX	34.36 ± 4.41	42	Female	Left colon	3	3	MSI	LS	38
HROC113	HROC113	Patient	35.04 ± 0.369	41	Female	Right colon	3	3	MSI	LS	20
HORC415MET	HROC415Met1 T0 M4	PDX	38.42 ± 3.96	33	Male	Abdominal wall	3	4	MSI	LS	
HROC50	HROC50 T1 M5	PDX	47.94 ± 9.23	67	Female	Right colon	2	2	MSI	spMSI	
HROC80	HROC80 T1 M1	PDX	53.36 ± 7.63	72	Male	Right colon	2	3	MSI	spMSI	
HROC370	HROC370	Patient	57.09 ± 1.64	77	Female	Right colon	2	1	MSI	spMSI	
HROC131	HROC131 T0 M3	PDX	64.79 ± 2.03	75	Female	Right colon	3	3	MSI	spMSI	
HROC126	HROC126	Patient	67.59 ± 5.71	58	Female	Rectum	2	3	MSS	spSTD	
HROC159	HROC159 T2 M4	PDX	68.2 ± 6.02	78	Female	Right colon	2	2	MSI	spMSI	
HROC324	HROC324	Patient	68.34 ± 6.02	55	Female	Right colon	3	4	MSI	LS	37
HROC112MET	HROC112Met1 T0 M2	PDX	70.36 ± 6.18	79	Female	Liver	2	4	MSS	CIMP-H	66
HROC183	HROC183	Patient	72.63 ± 6.06	59	Female	Right colon	3	3	MSS	CIMP-H	38
HROC285	HROC285 T0 M2	PDX	74.04 ± 9.25	30	Female	Left colon	2	4	MSI	LS	25
HROC252Tu2	HROC252Tu2 T2 M2	PDX	75.08 ± 1.02	45	Male	Sigmoid	3	2	MSI	LS	35
HROC217	HROC217 T1 M2	PDX	85.55 ± 14.06	38	Female	Left colon	2	4	MSS	spSTD	44
HROC300	HROC300 T2 M1	PDX	88.2 ± 10.75	73	Male	Rectum	2	4	MSS	CIMP-H	70

Supplementary Table 1. Information of the 21 HROC cell lines, patient characteristics, and clinical-pathological background.

Cell line	BioSample ID
HROC87	BioSample: SAMEA5820600; SRA: ERS3609190
HROC212	BioSample: SAMEA115337558; SRA: ERS18339074
HROC24	BioSample: SAMN47449425; SRA: SRS24413664
HROC383	BioSample: SAMN47449426; SRA: SRS24413665
HROC257	BioSample: SAMEA115337560; SRA: ERS18339076
HROC315	BioSample: SAMN44335383; SRA: SRS22934986
HROC113	BioSample: SAMEA115337552; SRA: ERS18339068
HROC324	BioSample: SAMEA115337566; SRA: ERS18339082
HROC112Met1	BioSample: SAMEA5820589; SRA: ERS3609179
HORC183	BioSample: SAMEA115337557; SRA: ERS18339073
HROC285	BioSample: SAMEA5820595; SRA: ERS3609185
HROC252Tu2	BioSample: SAMN44335382; SRA: SRS22934985
HROC217	BioSample: SAMN44335381; SRA: SRS22934984
HROC300	BioSample: SAMEA115337564; SRA: ERS18339080

Supplementary Table 2. Accession details for transcriptomic data.

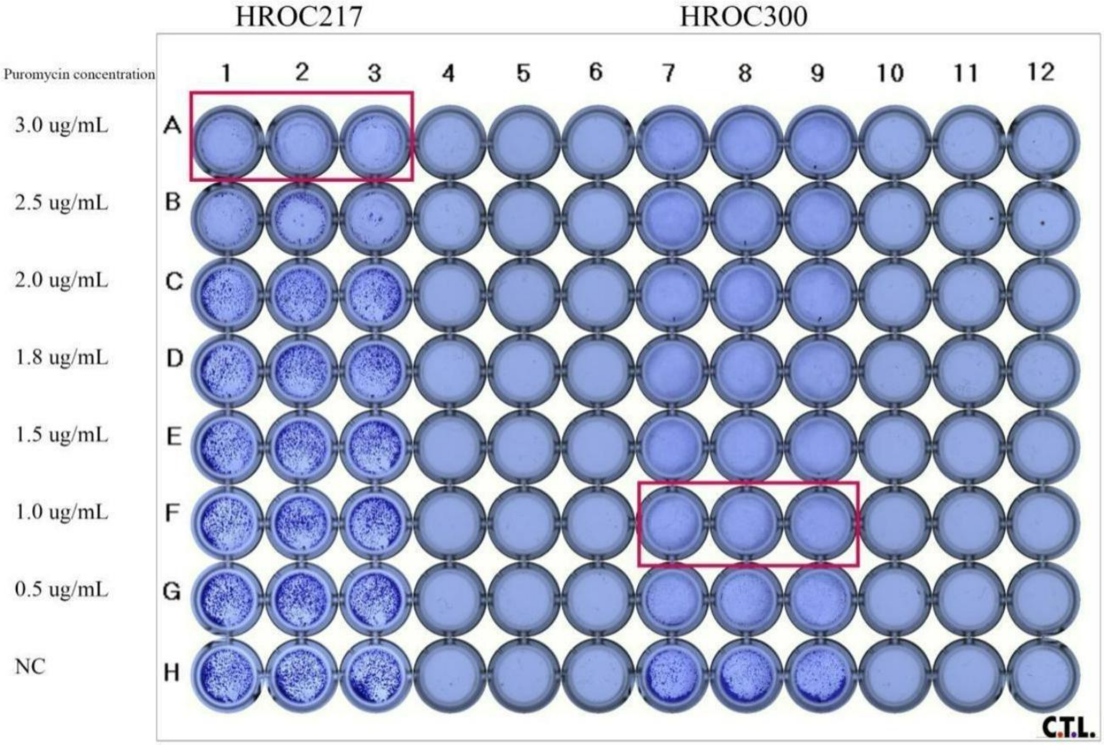

Supplementary Figure 3. Determination of optimal puromycin selection concentrations for HROC217 and HROC300.

Reagents for WB	Catalog Number	Manufacturer	Country	Dilution concentration
**Prestained Protein Marker**	PL00001	Proteintech	UK	5 μL
	PL00002	Proteintech	UK	5 μL
**Primary Antibodies**				
GAPDH Rabbit Polyclonal Antibody	TA890003	OriGene Technologies GmbH	Germany	1:2000
NAALADL1 Recombinant Rabbit Monoclonal Antibody	MA5-29434	Thermo Fisher Scientific Inc	Germany	1:1000
Caspase 3/p17/p19 Rabbit Polyclonal Antibody	19677-1-AP	Proteintech	UK	1:500
Vinculin Rabbit Polyclonal Antibody	26520-1-AP	Proteintech	UK	1:50000
Acetyl-α-Tubulin (Lys40) Antibody	3971	Cell Signaling Technology	Germany	1:500
**Secondary Antibody**				
StarBright Blue 520 Goat Anti-Rabbit	12005870	BIO-RAD	Germany	1:2000

Supplementary Table 3. Detailed information and dilution concentrations of antibodies used.

Figure 6A

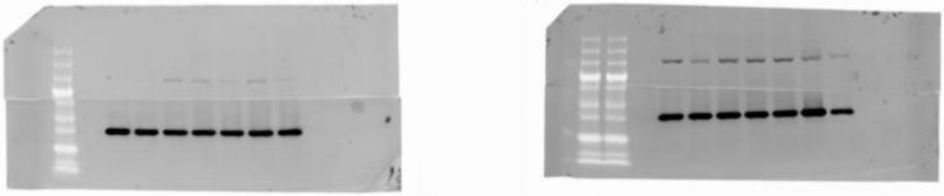

Figure 6B

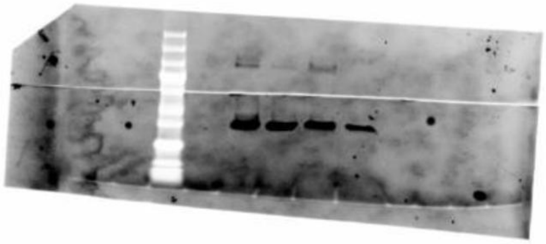

Figure 8F

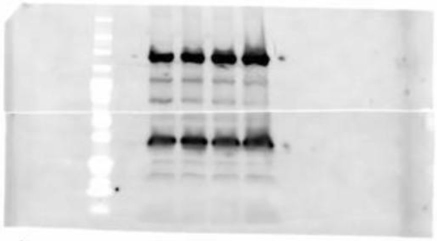

Figure 8G

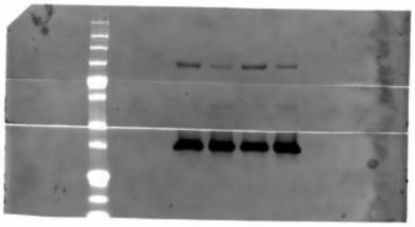

Supplementary Figure 4: Full blot of WB.
